# Supplementary material for: An improved reference of the grapevine genome reasserts the origin of the PN40024 highly homozygous genotype
Source: G3 (Bethesda). 2023 Mar 26;13(5):jkad067. doi: 10.1093/g3journal/jkad067 (PMC10151409; doi:10.1093/g3journal/jkad067)
Supplement: jkad067_Supplementary_Data [file jkad067_supplementary_data.zip › File_S2_G3-2023-404073.docx]

**Table S1** – Stranded V. vinifera RNA-seq data.

| **ENA Project/Run** | **Tissue** | **Treatment/ Note** |
| --- | --- | --- |
| PRJEB11405 | Wood | *Phaeomoniella chlamydospora infected* |
| SRX11034950 | Berries | ‘Cabernet Franc’ |
| SRX11034951 | Berries | ‘Cabernet Franc’ |
| SRX11034952 | Berries | ‘Kishmish Vatkana’ |
| SRX11034953 | Berries | ‘Kishmish Vatkana’ |
| SRX11034955 | Berries | ‘Rkatsiteli’ |
| SRX11034956 | Berries | ‘Rkatsiteli’ |
| SRX11034957 | Berries | ‘Sangiovese’ |
| SRX11034958 | Berries | ‘Sangiovese’ |
| SRX11034959 | Berries | ‘Savagnin Blanc’ |
| SRX11034960 | Berries | ‘Savagnin Blanc’ |
| SRX11034961 | Tendrils | ‘Cabernet Franc’ |
| SRX11034962 | Tendrils | ‘Cabernet Franc’ |
| SRX11034963 | Tendrils | ‘Kishmish Vatkana’ |
| SRX11034964 | Tendrils | ‘Kishmish Vatkana’ |
| SRX11034966 | Tendrils | ‘Rkatsiteli’ |
| SRX11034967 | Tendrils | ‘Rkatsiteli’ |
| SRX11034968 | Tendrils | ‘Sangiovese’ |
| SRX11034969 | Tendrils | Sangiovese’ |
| SRX11034970 | Tendrils | ‘Savagnin Blanc’ |
| SRX11034971 | Tendrils | ‘Savagnin Blanc’ |
| SRR9301839 | Leaf | ‘Cabernet Franc’ |
| SRR9301841 | Leaf | ‘Savagnin Blanc’ |
| SRR9301842 | Leaf | ‘Savagnin Blanc’ |
| SRR9301844 | Leaf | ‘Sangiovese’ |
| SRR9301845 | Leaf | ‘Kishmish Vatkana’ |
| SRR9301847 | Leaf | ‘Rkatsiteli’ |
| SRR9301848 | Leaf | ‘Cabernet Franc’ |
| SRR9301849 | Leaf | ‘Kishmish Vatkana’ |
| SRR9301850 | Leaf | ‘Sangiovese’ |
| SRR9301859 | Leaf | ‘Rkatsiteli’ |
| SRR9301860 | Leaf | ‘Sangiovese’ |

**Table S2** – Unstranded V. vinifera RNA-seq data.

| **ENA Project/Run** | **Tissue** | **Treatment/ Note** |
| --- | --- | --- |
| PRJNA168987 | Leaf | Trichoderma-induced *Plasmopara viticola* resistance |
| SRR3046429 | Berry skin | - |
| SRR3046438 | Berry skin | - |
| SRR3056885 | Berry | - |
| SRR3056886 | Berry | - |
| SRR3056888 | Berry | - |
| SRR3056889 | Berry | - |
| SRR5435969 | Leaf | *Plasmopara viticola* infection; Time series |
| PRJNA433195 | Berry | Full length cDNA included |
| PRJNA448744 | Flower | except SRR6951682 & SRR6951683 |
| PRJNA528770 | Leaf | except SRR8775073, SRR8775074, SRR8775075 |

**Table S3** – Datasets and weights used for data combination with EvidenceModeler.

| **Class** | **Dataset** | **Weight** |
| --- | --- | --- |
| Transcript | Stranded Transcriptome Assembly | 10 |
| Transcript | Unstranded Transcriptome Assembly | 8 |
| Protein | *A. thaliana* | 1 |
| Protein | Eudicotyledons SwissProt | 2 |
| Protein | Eudicotyledons OrthoDB10 v1 | 1 |
| Protein | Vitales | 2 |
| *Ab initio* prediction | GeneID | 1 |
| *Ab initio* prediction | SNAP | 1 |
| *Ab initio* prediction | GlimmerHmm | 1 |
| *Ab initio* prediction | BRAKER2 | 1 |
| Other prediction | VCost.v3 liftover | 5 |

**Table S4** – Gene identifier nomenclature and transfer. PN40024.v4 genes with a valid reciprocal best BLAST hit with a VCost.v3 gene were named according to the VCost.v3 gene, novel genes received the prefix ‘04’ at the start of the gene number and genes predicted for alternative heterozygous sequence regions received the suffix ‘_alt’.

|  | **ID** | **Alias** |
| --- | --- | --- |
| **Gene** | Vitvi02g01736 | VIT_00s0341g00010, LOC100264826, Vitvi00g00481 |
| **Novel Gene** | Vitvi01g**04**001 | - |
| **ALT Gene** | Vitvi02g04116_alt | - |
| **mRNA** | Vitvi01g04001_t001 | - |
| **CDS** | Vitvi01g04001_t001.cds1 | - |
| **Exon** | Vitvi01g04001_t001.exon1 | - |
| **5’ UTR** | Vitvi01g04001_t001.utr5p1 | - |
| **3’ UTR** | Vitvi01g04001_t001.utr3p1 | - |

**Table S5** – Assembly and anchoring statistics for the second allelic version, ALT, of PN40024.v4. For the anchoring of the ALT scaffolds, a total of 2,326 markers were used from the six Canaguier’s maps, in addition to 5,866 and 5,832 SNP markers from the ‘Riesling’ and the ‘Gewurztraminer’ GBS maps, respectively, to anchor these scaffolds.

| **SCAFFOLDS** | **Numb. Scaf.** | **Min size [bp]** | **Avg size [kb]** | **Median size [kb]** | **L50** | **N50 [Mb]** | **Max size [Mb]** | **Sum [Mb]** | **Numb. Ns** | **GC [%]** |
| --- | --- | --- | --- | --- | --- | --- | --- | --- | --- | --- |
| **ALT** | 485 | 542 | 955 | 21 | 24 | 6.50 | 15.22 | 463.03 | 1,675,751 | 34.4 |
| **Anchored ALT** | 166 | 1,085 | 2,749 | 1,496 | 24 | 6.50 | 15.22 | 456.28 | 1,553,419 | 34.4 |

**Table S6** – Comparison of RNA-Seq read alignments to the PN12X.v2 and PN40024.v4 assemblies and assignment to VCost.v3 and PN40024.v4.1 gene predictions.

|  |  |  | **PN12X.v2 - VCost.v3** | | **PN40024.v4 - PN40024.v4.1** | |
| --- | --- | --- | --- | --- | --- | --- |
| **Variety** | **Sample BBCH77** | **MM Seqs** | **Aligned [%]** | **Assigned [%]** | **Aligned [%]** | **Assigned [%]** |
| **‘Sangiovese’** | SRR1631822 | 31.2 | 92.1 | 87.9 | 92.3 | 90.3 |
|  | SRR1631823 | 33.3 | 92.2 | 88.3 | 92.4 | 90.9 |
|  | SRR1631824 | 32.3 | 92.3 | 88.3 | 92.5 | 90.9 |
| **‘Barbera’** | SRR1631834 | 40.7 | 91.4 | 87.5 | 91.5 | 90.2 |
|  | SRR1631835 | 37.2 | 90.8 | 87.6 | 90.9 | 90.2 |
|  | SRR1631836 | 43.6 | 91.4 | 87.0 | 91.5 | 89.6 |
| **‘Refosco’** | SRR1631858 | 39.2 | 91.6 | 88.1 | 91.6 | 91.1 |
|  | SRR1631859 | 36.7 | 91.8 | 87.8 | 91.8 | 90.8 |
|  | SRR1631860 | 43.8 | 91.2 | 88.2 | 91.3 | 91.1 |
